# POFBG-Embedded Cork Insole for Plantar Pressure Monitoring

**DOI:** 10.3390/s17122924

**Published:** 2017-12-16

**Authors:** Débora Vilarinho, Antreas Theodosiou, Cátia Leitão, Arnaldo G. Leal-Junior, Maria de Fátima Domingues, Kyriacos Kalli, Paulo André, Paulo Antunes, Carlos Marques

**Affiliations:** 1 Department of Physics & I3N, University of Aveiro, Campus Universitário de Santiago, 3810-193 Aveiro, Portugal; deboravilarinho@ua.pt (D.V.); catia.leitao@ua.pt (C.L.); fatima.domingues@ua.pt (M.F.D.); pantunes@ua.pt (P.A.); 2 Nanophotonics Research Laboratory, Cyprus University of Technology, Limassol 3036, Cyprus; theodosiou.antreas@gmail.com (A.T.); kkalli@cytanet.com.cy (K.K.); 3 Instituto de Telecomunicações, Campus Universitário de Santiago, 3810-193 Aveiro, Portugal; 4 Telecommunications Laboratory, Electrical Engineering Department, Federal University of Espírito Santo, Fernando Ferrari Avenue, Vitoria 29075-910, ES, Brazil; arnaldo.leal@aluno.ufes.br; 5 Centro de Automática y Robótica, CSIC-UPM, Ctra. Campo Real, Arganda del Rey, 28500 Madrid, Spain; 6 Instituto de Telecomunicações and Department of Electrical and Computer Engineering, Instituto Superior Técnico, University of Lisbon, 1049-001 Lisbon, Portugal; paulo.andre@ist.utl.pt

**Keywords:** polymer optical fiber, gait plantar pressure, CYTOP, physical rehabilitation, optical fiber sensors, insole fiber Bragg gratings network

## Abstract

We propose a novel polymer optical fiber (POF) sensing system based on fiber Bragg gratings (FBGs) to measure foot plantar pressure. The plantar pressure signals are detected by five FBGs, in the same piece of cyclic transparent optical polymer (CYTOP) fiber, which are embedded in a cork insole for the dynamic monitoring of gait. The calibration and measurements performed with the suggested system are presented, and the results obtained demonstrate the accuracy and reliability of the sensing platform to monitor the foot plantar pressure distribution during gait motion and the application of pressure. This architecture does not compromise the patient’s mobility nor interfere in their daily activities. The results using the CYTOP fiber showed a very good response when compared with solutions using silica optical fibers, resulting in a sensitivity almost twice as high, with excellent repeatability and ease of handling. The advantages of POF (e.g., high flexibility and robustness) proved that this is a viable solution for this type of application, since POF’s high fracture toughness enables its application in monitoring patients with higher body mass compared with similar systems based on silica fiber. This study has demonstrated the viability of the proposed system based on POF technology as a useful alternative for plantar pressure detection systems.

## 1. Introduction

Optical fiber sensors present advantages over conventional electronic and electromechanical sensors. These advantages include compactness, lightweight, data multiplexing capabilities, electrical isolation, electromagnetic field immunity, and biocompatibility [1,2]. For these reasons, optical fibers sensors have been used in medical applications, structural health monitoring, and industrial applications to measure parameters such as temperature [3], strain [4], humidity [5], refractive index [6], and pressure [7], among other physical quantities [8,9].

Silica optical fiber (SOF) sensors are most often used due to their lower transmission losses and ease of connectorization; however, recent advances in polymer fiber processing and techniques for the inscription of fiber Bragg gratings (FBGs) [2,10] have extended the use of polymer optical fiber (POF) sensors as a good alternative to the conventional SOF sensors. POF sensors offer higher bending flexibility, greater operation range, higher strain limits, lower production cost, and higher robustness, properties that are required for optical sensors [2,11]. Compared with SOF sensors, POF sensors present the additional advantage of higher fracture toughness [2], a result of their Young modulus that is almost 25 times less than that for silica glass. Therefore, POFs with diameters close to silica can display higher sensitivity, such as microstructured POFs in strain [12], stress and force measurements [13], pressure [14], and acoustic wave detection [15]. 

In order to achieve specific and tailored characteristics, the material properties of polymers can be chemically modified by adding different organic combinations, such as the perfluorinated compounds, resulting in the commercially known CYTOP (cyclic transparent amorphous fluoropolymers) POFs. In the CYTOP fiber, the carbon-hydrogen bonds are replaced with carbon-fluorine bonds to reduce fiber attenuation [16], reaching loss values comparable to silica fibers, enabling the use of this fiber at 1550 nm wavelengths. For this reason, standard and off-the-shelf optical components, originally designed for telecommunications purposes, can be applied, and this has the potential to lead to simpler and lower cost sensor systems compared with other types of polymer fibers. 

The POFs flexibility and non-brittle characteristics make these sensors clinically acceptable [17]. For this reason, numerous applications of POF sensors related to human safety have been proposed [18]. The applications related to human safety and health generally point towards the mitigation of the impairments provoked by the continuous aging of the population, which leads to the increased health risks and therefore the need for a close monitoring of elder citizens and patients. Although the field of e-Health has been experiencing great progress, it still faces challenges, which are generally related to the mobility compromise and lifestyle of the monitored patients. In order to overcome these challenges, mobile health monitoring devices need to provide low power consumption, size, and weight. Furthermore, high safety and privacy are also desirable. Therefore, the development of efficient solutions for healthcare sensor applications (regarding size, weight, and energy consumption) is an important research focus given the rapid technological advances in healthcare monitoring equipment, microfabrication processes, and wireless communication [19].

One of the key parameters monitored by researchers for biomedical applications is foot plantar pressure [19,20]. This parameter is an important indicator of the foot health condition and the gait pattern. Information regarding the condition of the spinal cord or regarding the evolution of foot ulcerations (in case of patients with diabetes) can be inferred from the plantar pressure distribution map. In the particular case of diabetes, patients tend to develop foot ulcerations, which can be detected by high/abnormal forefoot plantar pressure [21]. Hence, accurate and controlled evaluation of the plantar pressure is vital to reduce and eventually avoid the risk of such pathologies. Additionally, by mapping the ground reaction forces or pressures during gait it is possible to understand the effect induced in the body [19]. Further important information that can be inferred by the foot plantar pressure is the gait analysis, which is a systematic examination of body locomotion that can be used in the evaluation of pre-treatment for surgical decisions and post-operative assessment of patients [19]. The human gait is defined as the period of initial contact of the foot with the ground and lasts until the same foot touches the ground again [22]. A gait cycle is generally divided into two main phases: the stance and swing phases. The stance phase represents approximately 62% of the gait cycle for a person without gait abnormalities and is related to the period that the foot is in contact with the ground [23]. The stance phase starts with the initial contact (IC), at which point the foot touches the ground, followed by the maximum weight acceptance (MA), when the weight of the subject is on the heel region. Following this there is a rotation of the foot with the ankle as a pivot, and when the whole foot is in contact with the ground, the flat foot (FF) phase is reached. As the rotation continues, the heel loses contact with the ground, corresponding to the heel off (HO) phase. Finally, on the instant that the toe is off the ground, the toe off (TO) phase takes place and the swing phase starts [23].

Conventional technologies for plantar pressure assessment are mainly based on electronic or imaging devices. Several of the smart insole implementations, based on piezoresistive sensors and wireless data communication modules for walking gait rehabilitation monitoring, are reported in [24,25]. The important features often reported for these types of solutions are their high resolution data acquisition, robust and wireless communication, real time processing, and low power consumption [24]. Similar features may be anticipated for FBG sensors, as advances towards the miniaturization and integration of optical components are realized. However, fiber sensor devices present some drawbacks, including fragility, long-term instability, inconsistency, and excessive drift [26]. Additionally, their output is restricted to a small sensing area that requires the use of more sensors to monitor larger areas [26].

In order to overcome the limitations of conventional technologies, many scientific papers have been published reporting plantar pressure distribution monitoring techniques, and even though optical fiber sensing technology had already been used to monitor static plantar pressure values [27,28,29], the application of fiber Bragg gratings (FBGs) is rarely addressed [19,26]. To date, there is only a single report on the dynamic, continuous measurements during gait motion that was based on silica optical fiber technology [30]. In this respect, the achievement of this work regarding the continuous optical data acquisition during gait is of great significance for the field and where we have used polymer optical fiber. POF presents advantages over the conventional methods for plantar pressure assessment and advantages related to the material features when compared with the sensors designed with SOFs for plantar pressure assessment.

In this paper, we propose a fiber-optic sensors network based on CYTOP polymer optical fiber Bragg gratings (POFBGs) to monitor the plantar pressure. The architecture and characterization procedures are similar to the ones proposed in previous works employing SOFs [30], which make it possible to have a better comparison between the two technologies. The proposed system has the advantages of a simple architecture when compared with silica FBGs, a lower influence of temperature on the sensor response, and higher repeatability and sensitivity (near two times higher compared with SOF technology). Results also show good correlation between the four cycles in dynamic test achieving a root mean squared error (RMSE) of 160 kPa when all cycles are analyzed; this represents an error of less than 5%. Although FBGs in silica fibers are more available commercially, POFs can withstand lower curvature radius than silica fibers, where the minimum radius for the silica fiber is 10 mm [31] and for CYTOP is 5 mm [32]. POFBGs also provide the necessary resistance to the system during the gait movement in order to avoid damage or breakage, as can easily happen using silica optical fiber. Furthermore, POFs generally present strain limits of 10%, which can be increased if different polymers are employed [33]; whereas a realistic strain limit of 1% is typical for SOFs [34]. The higher resilience of POFs translates to a greater tolerance to impact loads, and this may enable its application to monitoring subjects with higher body weight than the ones that can be monitored with silica fibers, which is especially desirable, since the individuals with diabetes often have an increased weight [35]. Additionally, the obesity can be related to other pathologies that affect the gait cycle and the plant pressure distribution [36,37]. In summary, the proposed in-shoe POFBG system can be used in the measurement of human plantar pressure distribution to monitor and understand whether the foot posture needs to be corrected or not. To improve the life quality of physically impaired citizens, increase the mobility of elder citizens, as well as address the challenges mentioned before, an integrated “in-shoe” optical fiber sensor network, able to monitor health conditions by observing physiological parameters in the foot, is designed using POF technology.

## 2. POFBG-Embedded Insole Production and Characterization

Fiber Bragg Gratings are periodic (or quasi-periodic) perturbations of the refractive index of the fiber core, generally induced by UV radiation. These perturbations create a selective wavelength reflective structure that follows the Bragg condition. The sensing principle relies on the Bragg wavelength variation, which happens due to the effects of temperature and strain. Equation (1) presents the relation between the Bragg wavelength shift (∆*λ_B_*) with temperature and strain.

(1) $$\Delta \lambda_{B}=[(1-P_{e})\varepsilon +(\alpha +\zeta )\Delta T]\lambda_{B}$$

where *λ_B_* is the Bragg wavelength for the undisturbed situation, *ε* is the strain on the fiber, and *P_e_* is the photo-elastic constant. Furthermore, for the temperature influence on the wavelength shift, *α* is the thermal expansion coefficient, ∆*T* is the temperature variation, and *ζ* is the fiber thermo-optic coefficient. For the CYTOP fiber, the *P_e_* is approximately 0.041 [38], whereas, *α* and *ζ* are 74 × 10^−6^ K^−1^ and −50 × 10^−6^ K^−1^, respectively. Typical values of silica fibers for *P_e_*, *α,* and *ζ* are 0.22, 0.55 × 10^−6^ K^−1^, and 6.7 × 10^−6^ K^−1^, respectively. Since the silica fiber coefficients are lower than the ones of CYTOP, it is expected that CYTOPs present higher sensitivity to strain and temperature.

In this section, the production of the POFBG, as well as its characterization as a pressure sensor, is presented. The produced POFBGs are embedded on a natural cork sole that can be used as a fixed platform or as an in-shoe solution. 

### 2.1. POFBGs Production and Sensor Assembly

**Figure 1:**
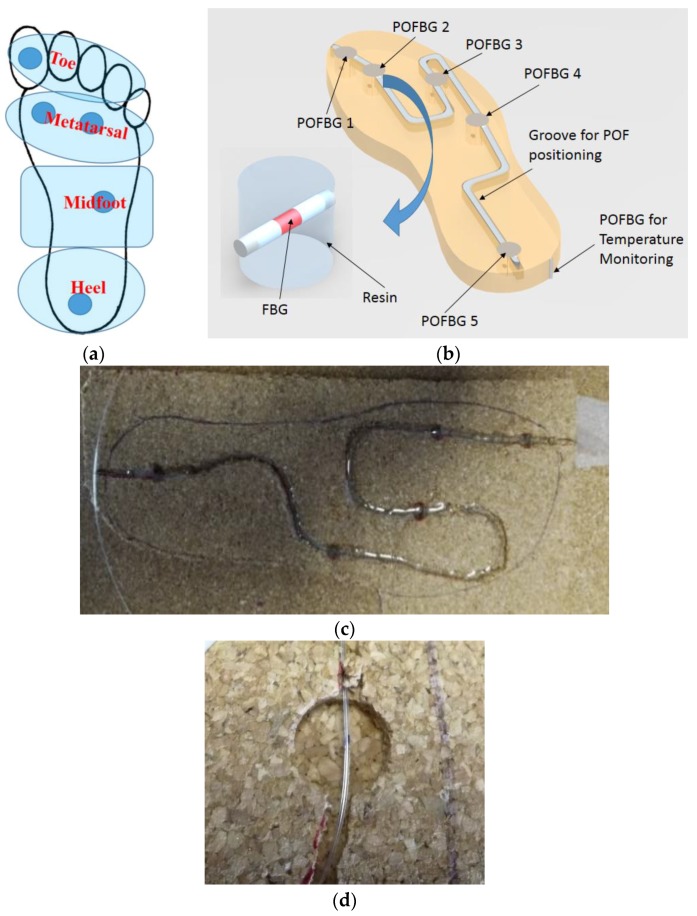
Schematic representation of: (**a**) Foot plantar area designation and sensing points distribution and (**b**) polymer optical fiber Bragg gratings (POFBG)-embedded cork insole. (**c**) Photograph of the POFBGs array embedded in the cork insole. (**d**) A zoom-in image of one sensing element.

The key areas of the foot for plantar pressure monitoring are presented in Figure 1a, namely heel, midfoot, metatarsal, and toe areas [19,20,39]. Five POFBGs sensors are distributed within these areas, embedded in the cork insole with 10 mm thickness. For the integration of the POFBGs in the sole, a groove with 2.5 mm depth and 2 mm width was caved in the cork sole. In the key areas mentioned, a cylinder with 5 mm depth and a diameter of 10 mm was also caved in the cork sole, as can be seen in Figure 1b–d. Although cork can display characteristics of plastic behavior following long periods of use, it has several advantages such as thermal isolation, malleability, and low Poison ratio (near zero) [40]. The latter prevents crosstalk between sensors, since the insole will isolate the five pressure sensing points. Furthermore, the low Poisson ratio can diminish the effects of shear stress during the gait on the plantar pressure assessment. The malleability property of the cork enables its application in different types of shoes, and the thermal isolation reduces the influence of the body temperature on the sensors response when compared to a gait cycle that generally takes about 1 s to occur [30]. The frequency with which the body temperature changes is much lower than the frequency of the gait cycle and therefore can be neglected. Considering the load pressure applied in the gait movement, the POFBGs were encapsulated in epoxy resin (Liquid Lens^TM^, Bedford, Bedfordshire, UK) cylindrical structures (10 mm diameter and 5 mm height). Each sensing element consists of such a cylindrical epoxy structure with the POFBG at the middle. The epoxy structure is mainly under axial strain when a force is applied on it. Such strain is transmitted to the embedded-FBG that leads to a wavelength shift proportional to the applied force. To compensate for any temperature change, an FBG temperature sensor [18] was incorporated in the insole in order to guarantee that the thermal isolation provided by the cork is effectively obtained and the FBG plantar pressure sensors are not affected by the body temperature, or any external temperature changes. Figure 1b presents a diagram of each sensor position on the cork insole. Figure 1c,d show a photograph of the POFBGs array embedded in the cork insole and a zoom of a POFBG in its sensing position.

**Figure 2:**
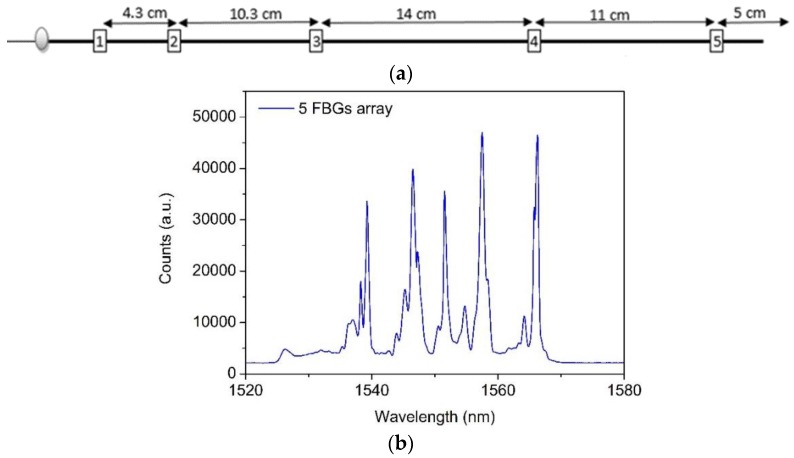
(**a**) Schematic demonstration of the physical distances between fiber Bragg gratings (FBGs) in the cyclic transparent amorphous fluoropolymers (CYTOP) fiber. (**b**) Reflected optical spectrum of the five-sensors FBG array inscribed in 120 µm core diameter, multimode, gradient index CYTOP fiber when illuminated with broadband light source and measured using a commercial FBG spectrometer.

The FBG sensors employed on the experiments were POFBGs inscribed in a gradient index multimode CYTOP fiber (Chromis Fiberoptics Inc., Warren, NJ, USA) with core diameter of 120 μm, a 20 μm cladding layer, and an additional polyester and polycarbonate over-cladding structure to protect the fiber, resulting in a total diameter of 490 μm [41]. The POFBG sensors were inscribed using the plane-by-plane femtosecond laser inscription method [42] and were physically separated, as shown in Figure 2a. The inscription setup used a femtosecond (fs) laser system (HighQ laser femtoREGEN) operating at 517 nm with 220 fs pulses duration to modify the material [42,43,44]. All the inscriptions were performed without removing the outer protection jacket, and the fiber samples were immersed in matching oil between two thin glass slides. The fiber samples were mounted on a nanometer accuracy air-bearing translation stage system (Aerotech) for accurate and controlled two-dimension motion during the inscription process. The laser beam was focused from above through a long-working-distance microscope objective ×50 (Mitutoyo) mounted on a third stage. By accurately controlling the focused beam, refractive index modifications can be induced in the centre of the fiber’s core without influencing the intermediate layers. The pulse energy at the exit of the laser was about 80 nJ, and the repetition rate was controlled using a pulse picker and set at 5 kHz for these particular inscriptions. The gratings’ period was close to 2.2 μm, resulting in the inscription of fourth order FBGs operating at the 1550 nm; small variations in the gratings periods (for different FBGs) set the operating wavelengths for FBGs in the array, in the range 1535 nm to 1580 nm. The length of the gratings was about 1.2 mm and the plane width was set at 15 µm in the center of the core to minimize the coupling between the higher order modes of the fiber and the grating. The reflection spectrum of the POFBG array right after fs inscription is shown in Figure 2b. The data was collected by a portable interrogation system powered by a battery, a miniaturized broadband optical ASE module (B&A Technology Co., As4500, Shanghai, China), an optical circulator (Thorlabs, 6015-3, Munich, Germany), and an optical spectrometer (Ibsen, I-MON 512E-USB, Farum, Denmark). The spectral full width at half maximum for each grating is between 0.6 and 1.0 nm.

### 2.2. Instrumented Cork Insole Characterization

**Figure 3:**
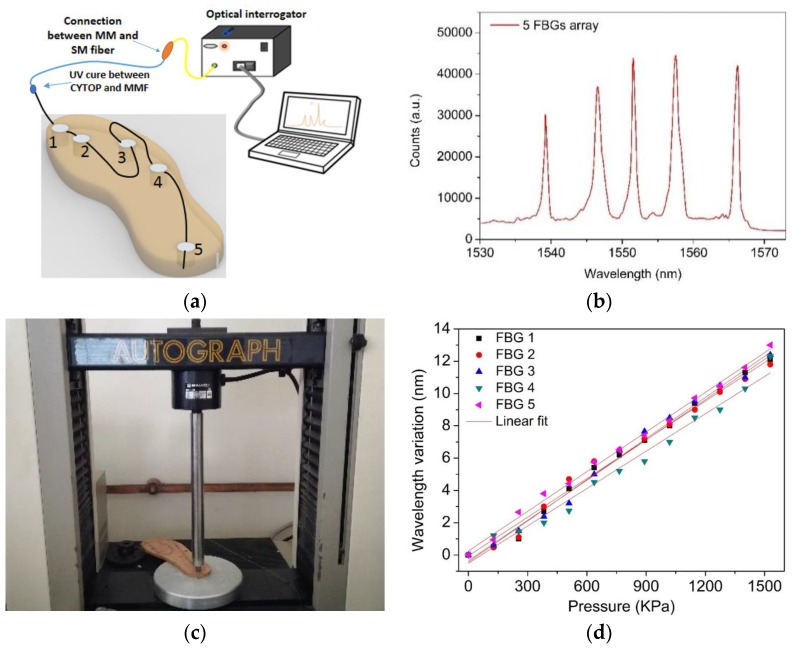
(**a**) Schematic representation of the monitoring system. (**b**) Reflected optical spectrum of the five-sensor FBG array after integrated in the cork insole; (**c**) photograph of the pressure testing platform with the cork insole; (**d**) calibration results of the POFBGs to pressure (points are the experimental values and lines refer to the linear fits, (0.982 < *R*^2^ < 0.994).

For the calibration and plantar pressure monitoring, the POFBG sensing network was connected to the portable interrogation system referred to above (composed of a battery, a miniaturized broadband optical ASE module (B&A Technology Co., As4500), an optical circulator (Thorlabs, 6015-3), and an optical spectrometer (Ibsen, I-MON 512E-USB)). The latter operates with a maximum acquisition rate of 960 Hz, and a wavelength resolution of 5 pm, and was used for the acquisition of the Bragg wavelength shift. Figure 3a shows the fixed platform monitoring system. To guarantee a stable connection and monitoring of the POFBG array, the polymer fiber was UV-glued with a silica multimode (62.5/125 µm fiber from Corning) fiber (MMF) for easier coupling, and then a silica single mode fiber (SMF) was spliced to the MMF in order to be compatible with our interrogation system. The spatial dimensions of the gratings in the center of the core excite the lower order modes of the MMF. In addition, the launch conditions linked with inscription parameters also play a role in the excitation of higher and lower order modes of the MMF [10]. In order to produce a grating array in the gradient index, multimode fiber, we must reduce the number of fiber modes coupling to each of the gratings. This is achieved by limiting the spatial extent of each FBG in the array to the central part of the core in the region where the gradient index profile peaks. We ensure that all gratings are in the same core location. In this way, when we launch light into the fiber we can excite all gratings simultaneously for one launch condition. Moreover, we are able to excite the strongest lower order modes as a result of the controlled grating spatial extent, and this leads to largely single peak POFBG spectra that are recovered together. There may be some mode coupling as a result of the changing launch, but there is predominantly one mode for signal processing. Figure 3b shows the reflection optical spectrum of the five-FBG array already integrated into the cork insole. The array of 5 FBG sensing elements were calibrated to different pressure loads ranging from 10 to 1500 kPa with 100 kPa steps (Shimadzu^®^ AGS-5Knd, Columbia, SC, USA), as shown in Figure 3c. The load sets were applied independently in each sensing point (from POFBG 1 to POFBG 5), using a probe with a diameter of 10 mm. During each load set, the reflected Bragg wavelength shift of the respective FBGs was acquired by the interrogation system, which was compared with the ratio between the applied force of the testing machine and the cross-sectional area of the epoxy resin cylinder. The calibration values for all sensing elements (POFBG 1 to POFBG 5) are presented in Figure 3d, in which the linear dependence of the Bragg wavelength shift with the applied pressure is evident. Table 1 summarizes all sensitivity coefficients for all POFBGs, and the RMSE of the POFBGs is compared with the load cell of the force platform. The characterization tests were made at a constant temperature of 22 °C. The temperature sensitivity of each POFBG is also presented in Table 1, for which the thermal characterization of the cork insole containing the POFBGs array was performed using a climate chamber. The temperature was raised from 22 °C to 52 °C in 5 °C steps. A 30 min’ stabilization time was given in each step to ensure thermal equilibrium. The mean thermal sensitivity of the CYTOP POFBGs is 18.42 ± 0.45 pm/°C, which is in agreement with [9].

We can make a comparison with the silica FBG-based insole presented in [30]. The proposed POFBG insole presents higher sensitivity when comparing all the 5 FBGs, the lowest sensitivity obtained for the POFBGs is 7.71 pm/kPa, the mean and standard deviation of all 5 POFBGs is 8.14 ± 0.31 pm/kPa; whereas, for the silica FBGs presented in [30], the lowest value is 0.89 pm/kPa with a mean value ± standard deviation of 2.51 ± 1.08 pm/kPa. In addition, the RMSE shows that the POF sensors present lower errors with the relative error, considering the 1500 kPa range of the test; the RMSE is <4%.

## 3. Force Platform and In-Shoe Applications of the Insole POFBG Plantar Pressure Sensors Network

### 3.1. Force Platform Application

**Figure 4:**
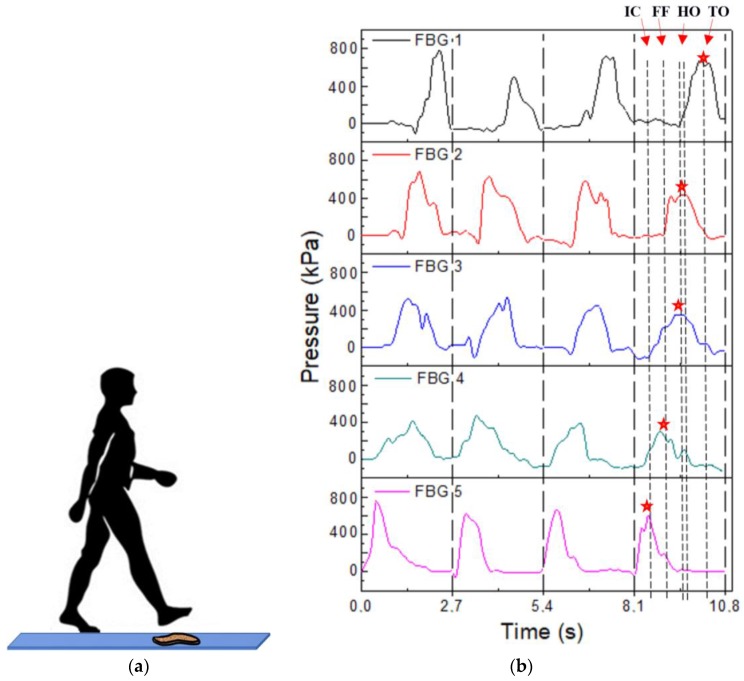
(**a**) Schematic diagram of the protocol implemented for the gait analysis using the fixed platform. (**b**) Pressure obtained during the four steps for the 5 POFBGs, in which the periods of stance phase (short dashed lines and star symbols) can be observed. IC: initial contact; FF: flat foot; HO: heel off and TO: toe off.

The pressure induced in the sensing elements during a normal gait movement was analyzed with the cork insole fixed on the ground, as shown in Figure 4a. The response of each pressure-sensing element during four gait cycles was recorded. The feedback of the platform to the displacement of the body center of mass (BCM) was also evaluated. This test was made with a female subject weighting 55 kg.

In Figure 4b, the acquired data is presented, from which it is possible to verify that the sensing network response is similar for the four passages, suggesting the human limits the repeatability of the sensors’ response. Furthermore, it is possible to confirm the activation time for each sensor, which is related to the stance phase of the gait cycle. The POFBG 5, located at the heel region, shows a pressure increasing at the beginning of the cycle (IC phase), and POFBG 4, 3, 2, and 1 are sequentially activated as the TO phase approaches. The dashed lines of the last cycle presented in Figure 4b represent the activation of each POFBG. The slight differences in each POFBG sensor for each passage are directly related to the differences in foot positioning on the platform during the tests.

**Figure 5:**
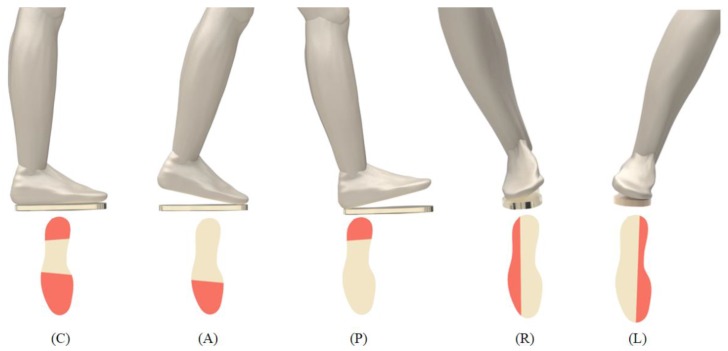
Schematic diagram of the protocol implemented for the analysis of the body center of mass (BCM) displacement and descriptive pressure increasing on the platform (the subject remained in each position for 3 s—the areas with increased pressure are coloured in red).

The second static test corresponds to the body center of mass displacements that are both in the body frontal plane by moving it forward and backwards, and in the sagittal plane by the subject moving the torso from the left to the right and vice-versa. For that purpose, the subject was asked to stand on the sensing platform, with one foot on the sensing area and the other leveled in the same horizontal plane, to execute a series of BCM movements (with about 3 s duration each). Figure 5 presents the protocol for the BCM displacement tests. The test starts with the subject standing still at the center position (C); the next step is moving the BCM to the front on the frontal plane to achieve the anterior (A) position, then back to the center (C) and then moving to the posterior (P) position. The movement on the frontal plane finishes at the center (C). The sagittal displacement is executed with the same procedure; however, in this case, the BCM is moved to the left (L) and right (R), instead of to the anterior (A) and posterior (P) positions. 

**Figure 6:**
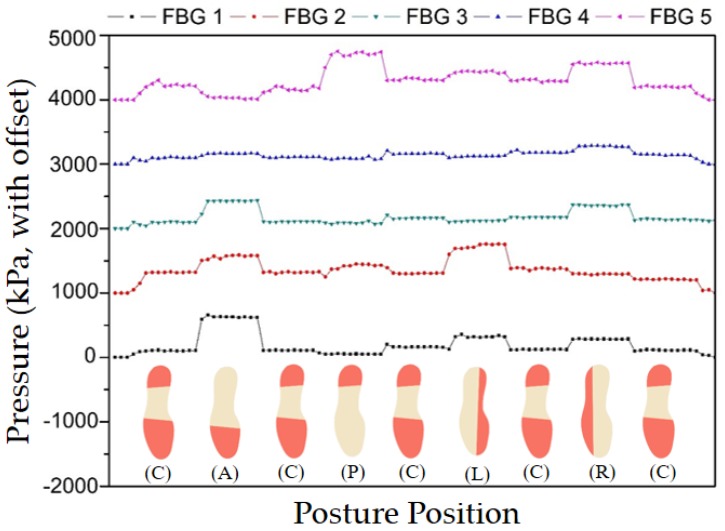
Representation of the pressures detected during the BCM displacements (the pressure increasing on each foot location is colored in red in the scheme).

During the protocol implementation, the Bragg wavelength shift induced in the sensing network was acquired, and the correspondent pressures were calculated. Figure 6 presents the response of each sensor during the different moments of the performed tests. 

For the tests on the frontal plane, an increase of the pressures registered by the sensors positioned in the metatarsal and toe areas is evident in the anterior movement, while the sensor placed in the heel section indicates a decrease of the pressure. On the other hand, during the posterior displacement of the BCM, the pressure at heel area is more accentuated, while the pressure values at the toe and metatarsal areas decrease. Regarding the centered position of BCM (C), the areas that are most often actuated in the platform are the metatarsal and midfoot areas, indicating that those zones experience greater ground pressure when sustaining the subject’s body weight whilst standing. It should be also noted that there was a small increase of pressure on the toe and heel regions. During the frontal tests, the behavior of the 5 POFBGs sensors are as anticipated, since the anterior and the posterior movements activate the heel and toe areas, represented by POFBGs 5 and 1, respectively. However, for body balance reasons, it is not possible to activate only the heel and toe regions, and therefore the sensors on the metatarsal and midfoot regions are also activated, namely POFBGs 2, 3, and 4, especially on the anterior movement, where the subject presents more difficulty to maintain her balance during the 3 s of the test. Furthermore, the sensors of the metatarsal and midfoot regions are more distant to the sensor on the heel region when compared to the distance of these sensors to the one at the toe region. For this reason, there is a higher isolation of the posterior movement, which explains the lack of increase on the pressure by POFBGs 2, 3, and 4 when the posterior position is taken.

Regarding the sagittal plane displacements, the sensors located at the extremities of the platform (FBG 2, FBG 3, and FBG 4) should be the ones presenting a higher variation of the pressure. In fact, it is noticeable that during such movements the sensor placed in position 2 records the increase of pressure when the BCM is displaced to the left, while the sensors placed in positions 3 and even 4 show a similar behavior when the BCM is moved to the right. There is also a lower activation of the sensor at position 1 (toe region) and at position 5 (heel region) due to the characteristic of the BCM displacement, which happens on the whole left and right regions of the foot. The results obtained on both sagittal and frontal plane movements show that the proposed sensor system is able to track the BCM movement on both planes with results similar to the results of reference [30].

### 3.2. In-Shoe Application

From the promising results of the cork insole positioned as a fixed platform during the performed tests, it becomes evident that the method implemented is an adequate solution for pressure monitoring during gait. Moreover, from the analysis of the pressures registered during the stance phase, it is also possible to infer and monitor the plantar pressures of individuals [29]. In order to perform a dynamic and autonomous pressure monitoring during gait, the insole having the five-FBG sensor network was adapted to a shoe, similar to the one developed in [30]. The foot plantar pressure fluctuation during gait is also induced in the instrumented cork sole. Such pressure oscillations resulted in shifts in the reflected Bragg wavelength, and these changes were monitored in the same way as in the previous tests.

**Figure 7:**
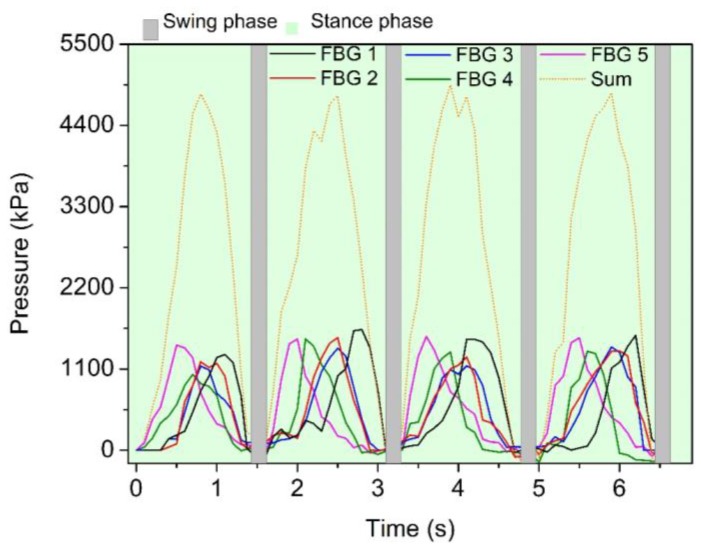
Pressure registered in the cork insole-sensing network for four gait cycles.

After converting the acquired wavelength shift, considering the sensitivity coefficient previously obtained (see Figure 3), the plantar pressure distribution induced in the cork sole over the gait movement was applied for the 5 POFBGs during 4 gait cycles, as shown in Figure 7. From the results obtained with the proposed insole sensor network, the repeatability of the data becomes clear, given the similar response of the sensors over the four gait cycles depicted in Figure 7. Also, it is possible to detect the sequence in which the sensors are activated (maximum amplitude registered), which was also in accordance with what is expected in a gait movement [19,30].

The POFBG 5 is activated first, at the beginning of the stance phase of the gait cycle, when the heel starts its contact with the floor. With the evolution of the cycle, POFBG 4 (located at the middle-foot and beginning of the metatarsal) is reasonably activated at the start of foot-flat stage during the single support. Following the gait movement, POFBG 2 and POFBG 3, located at the metatarsal positions, present a response at the forefoot contact during the middle of the stance phase. Finally, POFBG 1, located at the toe area, is activated at the toe-off moment, marking the end of the stance phase and the beginning of the swing phase of the gait cycle. The plantar pressure typical curve for the gait movement can be obtained by the addition of the 5 sensors’ feedback. The curve obtained represents the vertical ground reaction forces on the stance phase of the gait. On the swing phase, the foot loses contact with the ground the response of each POFBG is near to zero. Figure 7 presents the ground reaction forces obtained with the sum of the five POFBGs, showing a RMSE of 160 kPa between the four cycles, which represents an error of only 5% when compared with the average of the sum from the five POFBGs over four cycles. The description of the dynamic measurements was made to show that the proposed system is capable of detecting the subdivisions of the stance phase discussed in the introduction to this paper, which can aid in the detection of gait-related pathologies, such as spine anomalies, or foot ulcerations in patients with diabetes [30]. In addition, the detection of the gait phases can be applied to the controllers of wearable robots for gait assistance [45].

The results illustrated in Figure 7 show that our method is able to acquire an accurate gait pattern curve using CYTOP POFBGs-based sensing network, similarly to that previously reported in the literature using silica FBGs-based solution for the entire gait cycle analysis [30]. The main advantages obtained with POF technology when compared with silica fiber technology are the following: a much greater sensitivity considering the wavelength shift with the pressure variation (almost two times better than that reported in reference [30,46]), improved repeatability from the sum of each cycle presented in Figure 7, and higher flexibility. In addition, it is easier to handle the fiber incorporation into the cork sole, avoiding the comparatively easy breakage of silica fibers, since in many cases [30] it has its outer protection removed for the gratings inscription, which greatly increase its fragility. 

The temperature control sensor, a CYTOP FBG located in the cork insole, remains constant at 22 ± 0.2 °C (after the sensor is properly calibrated), validating the thermal isolation of the cork used for the instrumented sole production.

Finally, any limitations for the maximum allowable velocity that the system is capable of measuring are related to the material response and acquisition frequency of the interrogator. The epoxy resin was tested in frequencies higher than 1 kHz [47], whereas the interrogator presents an acquisition frequency of 960 Hz. Since the sampling frequency of 200 Hz is sufficient for acquiring the gait activities [19], the proposed system is able to cover the velocities employed in human gait. In addition, based on simple stress-strain relations [48], the proposed sensor system can, in principle, measure the plantar pressure of subjects with body mass higher than 200 kg, whereas, the sensitivity of each POFBG presented in Table 1 and the 0.5 pm resolution of the employed interrogator enable the detection of weights lower than 1 kg, which leads to a high dynamic range of the proposed insole.

## 4. Conclusions

The fast growth of mobile technologies has brought new insight into healthcare systems and practices, igniting the research and progress of the e-Health topic that has emerged with the field of Internet of Things (IoT). Our proposed and demonstrated system could easily include a wireless transceiver, capable of uninterrupted connection to a cloud-based monitoring application. The system could use a stand-alone wireless device dedicated to the monitoring design or a software application to be installed on a patient’s smartphone [49]. As mentioned above, the paper concentrates on the implementation and calibration of the CYTOP FBGs sensing network; to complete the remote plantar pressure monitoring platform, a mobile wireless gateway, responsible for the data process and wireless transmission to the decision centres, will be incorporated and explored very soon. This can be achieved with compact interrogators incorporated in the shoe or on the subject’s waist. A similar system is already presented in [46,49], with data processing and storage on a cell phone that sends data through a secured wireless connection. In conclusion, with the presented architecture, a framework for monitoring the health conditions of citizens and providing automated visual feedback using different state-of-the-art technologies (advanced optical fiber sensors technology, secured energy-efficient wireless broadband access systems, and smart actuators, among others) can be developed.

The results, using the CYTOP fiber, showed a good response when compared with previously reported solutions using silica optical fibers, with a mean sensitivity of 8.14 ± 0.31 pm/kPa, which is almost four times higher than the one obtained for the same application with silica FBGs (2.51 ± 1.08 pm/kPa), and good repeatability. A RMSE of 160 kPa was estimated comparing each gait cycle to show the sensor system repeatability, whereas the mean RMSE of 45.17 kPa that represents a relative error below 4% is obtained for each POFBG when compared to the reference measurement system of the testing machine; taking advantage of the polymer optical fiber benefits (high flexibility for instance), this is a viable solution for this kind of application. Such higher sensitivity leads to a higher wavelength shift of the POFBGs with the same pressure variation when compared to FBGs in silica fibers, which enable the application of low-cost interrogators with lower wavelength measurement resolution (about 5 pm) compared to its higher-cost counterparts (about 0.1 pm). Furthermore, POFs present higher fracture toughness and higher strain limits than SOF, which can enable its application for measuring the plantar pressure of individuals with higher body mass than the ones that the insole based on SOFs are capable of measuring. This higher sensitivity may also enable the use of lower cost interrogation setups, such as the ones with edge filters [50,51] that employ the beating between the grating and the filter with interrogation using photodetectors, allowing, at the same time, an increase in sensor portability. For the next step, improvements to the calibration will be realized by examining a large group of subjects with different physical gait characteristics in order to achieve an enhanced performance analysis.

## Figures and Tables

**Table 1:** Pressure sensitivities obtained for the sensor characterization.

FBG	Pressure Sensitivity (pm/kPa)	Temperature Sensitivity (pm/°C)	RMSE * (kPa)
1	8.31 ± 0.20	18.4 ± 0.42	36.59
2	7.99 ± 0.28	18.2 ± 0.47	54.47
3	8.51 ± 0.23	18.9 ± 0.41	42.76
4	7.71 ± 0.31	18.5 ± 0.49	62.81
5	8.20 ± 0.15	18.1 ± 0.45	29.22

* RMSE values are relative to a range of 1500 kPa.
